# Manufacturing with pluripotent stem cells (PSConf 2021): Key issues for future research and development

**DOI:** 10.1111/cpr.13301

**Published:** 2022-08-07

**Authors:** Glyn N. Stacey, Jingyi Cao, Baoyang Hu, Qi Zhou

**Affiliations:** ^1^ International Stem Cell Banking Initiative Barley Herts UK; ^2^ National Stem Cell Resource Center Chinese Academy of Sciences Beijing China; ^3^ Institute for Stem Cell and Regenerative Medicine Chinese Academy of Sciences Beijing China; ^4^ State Key Laboratory of Stem Cell and Reproductive Biology, Institute of Zoology Chinese Academy of Sciences Beijing China; ^5^ Beijing Institute for Stem Cell and Regenerative Medicine Beijing China; ^6^ University of Chinese Academy of Sciences Beijing China

## Abstract

Human pluripotent stem cells (hPSC) have the capability to deliver novel cell‐based medicines that could transform medical treatments for a wide range of diseases including age‐related degenerative disorders and traumatic injury. In spite of significant investment in this area, due to the novel nature of these hPSC‐based medicines, there are challenges in almost all aspects of their manufacturing including bioprocessing, characterization and delivery. The Chinese Academy of Sciences and the Chinese Society for Stem Cell Research have collaborated to create a new discussion forum called PSConf 2021 (Pluripotent Stem Cell Conference 2021), intended to promote exchanges in communication on cutting‐edge developments and international coordination in hPSC manufacturing. The PSConf 2021 addressed crucial topics in stem cell‐based manufacturing, including stem cell differentiation, culture scale‐up, product formulation and release. This report summarizes the proceedings and conclusions from the discussion sessions, and it is accompanied by publication of individual papers from the speakers at the PSConf 2021.

**Significance Statement:**

The PSConf 2021 meeting has brought together speakers and delegates from more than 20 countries in an informal discussion forum focusing on the manufacture of cell‐based medicines using hPSCs. The conference discussion sessions enabled an open exchange of information on the latest developments, ideas on key challenges and their potential solutions. It also captured the experiences and lessons learnt by professionals who had been in the field from the earliest applications of human embryonic stem cells, and presented a diverse range of new potential pluripotent stem cell‐based medicines that are now under development, with some already in clinical trials.

## Graphical abstract

Challenges in the manufacture of human plurtipotent stem cell‐based medicines.

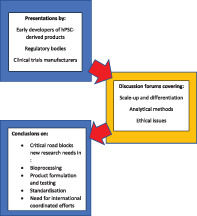

## 1 INTRODUCTION

Pluripotent stem cells present an exciting area of research due to their capability to recreate cells representative of all tissues of the human body, thus opening up an unprecedented avenue of research to develop accurate models of human tissue systems or provide cells for repair or replacement of diseased tissue. Since the discovery of human embryonic and induced pluripotent stem cell lines^1^, ^2^ there has been huge international investment in basic research to explore their potential in improving our understanding of cell biology and early development, in vitro diagnostic systems for drug discovery and safety testing, and regenerative cell‐based medicines. In more recent years, the potential for real applications of human pluripotent stem cells (hPSCs) in some of these fields has begun to be realised^3^, ^4^ and, in particular, independent monitoring of the development of hPSC clinical applications indicates that there are now more than 100 clinical trials for products manufactured using hPSCs.^5^

The Institute of Zoology (IOZ), Chinese Academy of Sciences (CAS) and the Beijing Institute for Stem Cell and Regenerative Medicine worked together with other Chinese and international stem cell networks (Appendix 1) to create an event (PSConf 2021, 21‐26th April 2021) hosted by the Institute for Stem Cell and Regeneration (ISCR), CAS, in Beijing. The aim of this meeting was to discuss the state of the art in hPSC‐based manufacturing and consider the pressing research needed to promote the development and implementation of stem cell‐based medicines.

The meeting programme captured the experiences of people who had been in the field from the earliest applications of human embryonic stem cells (hESCs) and also included a diverse range of new potential hPSC‐based medicines that are now under development, some already in clinical trials. The meeting, which attracted over 500 delegates from 23 countries, was composed of a series of three workshops that addressed a number of crucial topics for stem cell‐based manufacturing, including stem cell differentiation, culture scale‐up, product formulation and release. This report summarizes the proceedings and conclusions from the discussion sessions and is accompanied by publication of individual papers from the speakers at the PSConf 2021 meeting. It illustrates the great advances being made in the manufacture of hPSC‐based medicines, and provides a consensus perspective on the immediate needs for new research needed to resolve key road‐blocks and accelerate the development of therapeutic products from hPSC lines, for everyone in the field.

## 2 MANUFACTURING SCALE‐UP AND DIFFERENTIATION

In a welcome introduction, Professor Baoyang Hu (ISCR, CAS, China) officially opened the workshops and welcomed all delegates. He introduced the session by outlining the 21 hPSC‐based clinical studies that are now in progress in China, all of which had been registered with the Chinese regulator (NMPA) and www.clinicaltrials.gov. Prof Hu described the 11‐stage programme that each study was being subjected to, from in vitro and in vivo to clinical studies. Two products are at Phase II clinical trials, one product is progressing through the Investigational New Drug (IND) stage, and a further four are now at clinical research level. Therapeutic indications that had progressed to clinical studies ranged from neurodegeneration, to age‐related macular degeneration (AMD), cartilage damage, female reproductive diseases and fibrosis (both lung and uterine).

Dr Alex Zhang (Zephyrm Biotechnologies, China) presented an overview of the key issues from the perspective of an innovative pharmaceutical company and now an early independent developer of cell‐based medicines. He began by identifying top‐priority challenges that were particular to a cell‐based medicine:

- Safety in terms of in vivo distribution, tumorigenicity and immunogenicity/immunotoxicology,
- Efficacy in terms of generating the appropriate cell type for the intended indication, mechanism of action and obtaining the appropriate dose for patients,
- Establishment of a robust Quality Management System to assure reproducible products, with special attention to cell heterogeneity and sustained characteristics after industrial scale production.

Dr Zhang compared the manufacturing process of monoclonal antibodies with hPSC‐based manufacturing and indicated the common requirements of establishing a stable manufacturing cell line, cell banking, culture expansion, purification of hPSC‐derivative progenies and formulation of the final product. However, he noted the significantly greater complexity of bioprocessing hPSCs. Whilst he acknowledged that many labs could learn the hPSC culture technology, it was nevertheless challenging to translate and scale‐up the technology in a manufacturing environment. Furthermore, hPSCs were prone to appearance of genetic variants which may have significant yet hitherto ill‐defined implications for recipient patients. Dr Zhang outlined the manufacturing process to generate mesenchymal stromal‐like cells (M cells) from hESCs and identifieded the key road‐blocks as the development of stable culture conditions, 3D scale‐up technologies and automated on‐line monitoring during bioprocessing.

In order to generate robust and reproducible bioprocessing, Dr Zhang explained how he had used process analytical tools to identify those Critical Manufacturing Attributes (CMA), including requirements for critical reagents (e.g., nutrients/media, growth factors, microcarriers, cell seeding density) and process CMAs (e.g., cytokine profiles, glucose levels). This enables system modelling based on such process CMAs and Critical Process Parameters (CPP), such as the metabolic profiles and rate of changes in pH. In conclusion, Dr Zhang emphasized the crucial importance of high quality and informative Critical Quality Attributes (CQA) for the final product, including parameters for phenotypic identity, purity and potency. He saw the development of large‐scale bioreactor systems and the maintenance of genetic and phenotypic stability throughout the manufacturing process, as the most significant challenges remaining for the clinical utility of M‐cell products. This topic has also been explored in greater detail by Zhang and Stacey (2022).^6^

Dr Cedric Ghevaert (Wellcome Trust‐MRC Cambridge Stem Cell Institute and NHS Blood and Transplant, UK) presented on the development of a manufacturing process for a clinical trial based on hESC‐derived megakaryocytes being developed as part of the UK Regenerative Medicine Programme (https://www.ukrmp.org.uk/home-extended/mrc/). He explained that whilst this work involved the development of a cell type that had been used for many years, the use of a new and substantially different bioprocessing technology would require an almost complete rethinking for the whole process of product delivery and use. Dr Ghevaert outlined the complex multistage manufacturing process, with key intermediate active ingredient stages of megakaryocyte‐erythroid progenitors, megakaryocytes and platelets requiring a 100‐day process of directed differentiation based on a predefined sequence of environmental cues, including cytokines and feeder cells. He also described the additional work required to source raw materials of suitable quality for manufacturing and the subsequent optimisation of culture conditions. He explained the significant benefits in the optimisation of platelet yield that this process had delivered.^7^ He also emphasized that understanding the culture and differentiation process through process analytical tools, with a focus on those stages that were most challenging to develop into a GMP environment, had been key to progress towards a final manufacturing process.^8^ Dr Ghevaert outlined the significant work involved in the development of new potency assays including an in vitro thrombus formation and a laser‐induced injury model in NSG mice.^6^, ^8^ A major issue that the whole hPSC manufacturing field is facing today, is the selection of appropriate manufacturing cell lines. Dr Ghevaert explained the significant diversity that the UKRMP programme had discovered in the differentiation characteristics of different hESC lines. He concluded by stating that significant challenges remained in scale‐up, cost‐science‐based safety and quality standards, and achieving acceptability and adoption by healthcare providers. One advantage of hPSC‐derived platelet products was that they were generally considered to be more like traditional protein biologics, and not a cell therapy, as they were small cell fragments that were not nucleated. However, this regulatory perspective had also brought a focus on assuring a very high degree of product purity. To conclude, Dr Ghevaert reported early work for future developments to transition from 2D to 3D manufacturing processes using ‘sponges’ containing perfusion bioreactors and he also saw great possibilities for ‘supercharged platelets’ modified by TALENS gene editing to increase the levels of von Willibrand factor expression and product clotting performance.^6^, ^8^ It was clear from Dr Ghevaert's presentation that even hPSC‐derived products that appear quite simple, such as platelets, still require the resolution of complex biological challenges to deliver a reliable manufacturing process.

Dr Jane Lebkowski (Regenerative Patch Technologies LLC, USA) drew on her extensive history of involvement in various hPSC‐based product development programmes since the early 2000s. She said it was important to think beyond the immediate regulatory demands for the clinical trial and that it was important for product developers to include in their thinking the scalability and distribution for the anticipated patient community, as well as regulatory considerations at the post‐registration stage, and the costs of the final product. In particular, she emphasized the importance of establishing a cell banking system at a scale that could service the whole life cycle of the product. She concurred with many of the previous comments and pointed out that the issue of scalability would be the most crucial to enable ready access for national populations. Dr Lebkowski emphasized that identifying valuable CQAs was crucial for all later stages of manufacture, and particularly the scale‐up and reagent improvement. She thought it was important to assess a range of characteristics during process development, including the use of secretome and transcriptome analytics. Regarding CPPs, in Dr Lebkowski's experience, it was important to focus on raw materials, any holding steps (e.g., banked intermediate differentiated cultures) and any scaffold used to formulate the final product. She also went on to outline the critical requirements needed to enable effective storage and shipment of the final product, which included storage, formulation, stability, shelf‐life, shipment methods and handling at the clinical trial site. Dr Lebkowski concluded by emphasizing the importance of developing a good understanding of the product CQAs, and the establishment of robust assays for CQAs that would enable monitoring throughout the process development. She also urged product developers to plan for suitably scaled production that would meet the patient population needs at a cost that would enable commercial development.

Dr Kapil Bharti (National Eye Institute, National Institutes for Health, USA) reflected on a key challenge in his programme to manufacture a retinal pigmented epithelium (RPE) product using iPSC lines derived from the patients, which was that each product was derived using a different starting material. He reported that this issue had meant that IND‐enabling studies for autologous products in the US require manufacturing process validation before each clinical product can be manufactured, whereas this was not the case for allogenic products. Dr Bharti went on to describe the challenge of assuring the purity of cell products and illustrated the challenge of validating the absence of undifferentiated stem cells by flow cytometry. In the case of Dr Bharti's study, the use of a Convolutional Neural Network technique had shown that artificial intelligence approaches could facilitate reproducible evaluation of RPE cell batches and could detect morphological variations not seen using traditional microscopical observation by experienced operators. He also emphasized the need for functional assays such as electrophysiological analysis of RPE‐monolayers. Dr Bharti also discussed the challenge of assuring an acceptable low level of undifferentiated hPSCs, by reflecting on his laboratory's experience in achieving release criteria for the detection of hPSC markers of around 0.5% and demonstrating detection levels of 0.01% through spiking studies. Dr Bharti went on to describe pre‐clinical GLP studies of dose requirements, toxicity, biodistribution and tumorigenicity in rats for a number of different patient‐derived iPSCs. Furthermore, in order to give such safety assurances, he believed that preclinical testing and investigation of biodistribution and safety in immunocompromised animals may need to be performed.

The issue of elimination of reprogramming vectors was raised during the Q&A, and Dr Bharti responded that this was confirmed by qPCR at the working cell bank stage which was typically passage 10 following isolation of the iPSC clone.

Prof Benjamin Reubinoff and Ms. Shelley Tannenbaum (Hadassah University Hospital, Israel) reviewed some key regulatory issues they had experienced in manufacturing hESC‐based therapies in Israel. In particular, they reflected on the huge challenge of achieving cell numbers to deliver 10^9^–10^12^ cells for each patient, an equivalent number for pre‐clinical in vivo studies, and then reproducible batches of hPSCs for multiple individual patients in ongoing clinical trials. In their hands, traditional cell culture solutions in 2D formats had proven to be of limited value for hESCs derived, expanded and differentiated under xeno‐free conditions,^9^ and they had developed approaches for derivation, expansion and differentiation of hPSCs all in 3D suspension format, so that it would be more amenable to large scale operations.^10^ These yielded reproducible batches of hESC clusters that could be converted to neural cell spheroids. They showed that 3D dynamic culture technology, such as the use of stirred tank bioreactors at various stages of manufacture, yielded high cell numbers that were sufficient for multiple clinical doses. In their ongoing research, Prof. Reubinoff and Ms. Tannenbaum presented various options for suspension bioreactors, and concluded that a vertical stirred tank system (PBS Biotech, Inc., Vertical‐Wheel™ Bioreactor) provided uniform distribution of hydrodynamic forces, thus, reducing shear stress and yielded cell aggregates of uniform size, which sustained a diploid karyotype and pluripotent characteristics. Furthermore, this system gave significantly higher cell number yields compared to typical horizontal‐blade spinner‐type stirred tanks systems.^11^ Their work had also revealed the key factors in successful high yield suspension culture, that include selected seeding density, careful timing of media changes to control nutrient and metabolite levels, and selection of suitable tools to increase culture attachment surface area (e.g., microbeads, aggregates, hydrogels). Prof. Reubinoff and Ms. Tannenbaum addressed regulatory concerns regarding such bioreactor culture systems including culture stability (genetic and phenotypic), cell purity, bioprocessing validation and quality of the final formulation in the presence of particulates.

For the future of manufacturing, they concluded that further developments would be required, including a shift from open systems to closed and automated ones, an adoption of additional in‐process checkpoints including intermediate cell banks, and automated and computerized final formulation and packaging systems. Prof Reubinoff and Ms Tannenbaum also explored some of the topics in greater detail, which is published in tandem with this meeting report.^12^

## 3 ANALYTICAL METHODS AND RELEASE

Prof Roger Barker (University of Cambridge and Addenbrookes hospital Brain Repair Centre, UK) led the discussion by explaining the importance of selecting a disease and a relevant patient group within that disease entity, that would be likely to benefit most from a cell‐based therapeutic approach. In the case of Parkinson's Disease, he described the two approaches; (i) to actually repair the dopaminergic cellular network (using cell or gene therapy) or (ii) arrest the disease process or slow it down (e.g., anti‐inflammatory treatments, alpha‐synuclein based treatments). He described his work, supported by an EU consortium, focusing on allogenic cell replacement with hPSC ventral mid‐brain progenitor cells as the STEM‐PD product to replace midbrain A9 dopaminergic neurones. He emphasized the importance of selecting a suitable production cell line, by assessing its traceability and the whole process of its development. Dr Barker also reviewed the pros and cons of hESC‐ versus iPSC‐lines and concluded that whilst iPSCs had a less controversial ethical status and held the promise of HLA‐matched therapy, they also carried more concerns regarding tumorigenicity and long‐term patient safety given how they are generated. He outlined the importance of reagents and protocols that met the requirements of GMP manufacturing and the extensive GLP‐accredited testing at production cell bank and final product levels that were essential to meet regulatory acceptability. Prof. Barker also reviewed the extensive work that had been needed for bioprocessing protocol development and the in vivo and in vitro preclinical studies which had taken 6 years to complete. He looked forward to the conclusion of the STEM‐PD clinical trial programme that was currently moving to deliver a clinical trial in 2022.

Following Prof Barker's talk, discussion with delegates included consideration of the need for CE marking for devices in the EU, which may include certain equipment, reagents and testing systems used for hPSC manufacturing. It was clear that CE mark approval status would be required in the EU for such products used after the clinical trial stage and may need considerable effort and cost to achieve approval. The variation in product purity was also discussed and may require careful purification of therapeutic cells. Prof. Barker said that the culture system his group was using selected against residual stem cell populations. He also explained that in his collaboration with Prof. Malin Parmer (Lund University, Sweden), the Lund group had qualified the purity of the final product using antibody and molecular analysis of cell populations.

The issue of preclinical assessment of implanted cell biodistribution was addressed in detail by Prof. Patricia Murray (University of Liverpool, UK) whose centre had utilized a portfolio of novel imaging approaches.^13^ In this presentation, Prof. Murray focused on non‐invasive imaging techniques for studies of MSCs and hPSC‐based products. In order for biodistribution studies to yield useful data, Prof. Murray considered that it was important to develop techniques that would enable better understanding of cell viability, but also the proliferative state and differentiation status. This would hopefully enable verification that therapeutic cells can access and populate the intended target site, and exclude potentially hazardous distribution and accumulation of cells at unintended sites. Tracking cells in this way would hopefully enable an important understanding of safety, efficacy and mode of action of the cell‐based medicine. Prof. Murray emphasized that this knowledge is key to determination of the risk‐benefit ratio of the therapeutic, and facilitate optimisation of product safety and efficacy. She also described a model for whole body biodistribution studies in mice using intra‐cardiac inoculation of mesenchymal stromal cells that were genetically modified to express firefly luciferase and ZsGreen reporters. These studies had shown that bioluminescence was highly sensitive and gave data on cell viability and proliferation, but suffered from poor spatial resolution and yielded only planar images. Prof. Murray also overviewed other work using magnetic resonance imaging to visualize mesenchymal stromal cells labelled with iron‐oxide particles, which gave high spatial resolution that allowed analysis of intra‐organ biodistribution, but did not indicate cell viability. She reported a recent collaboration with Prof. Roger Barker at the University of Cambridge, to use a combination of bioluminescence and iron‐oxide labelling tracking methods followed by histological investigation to assess the fate of hESC‐derived dopaminergic neuron progenitors in rats.^13^ They are now assessing the use of materials that enable sustained release of specific growth factors such as GDNF and BDNF (PODS®, developed by the UK company Cell Guidance Systems), to explore their ability to promote the viability and differentiation of dopaminergic progenitors following implantation.

In the discussion following Prof. Murray's presentation, she was asked about the availability of the PODS® that enabled sustained release of specific growth factors. Prof. Murray said that these were commercially available from Cell Guidance Systems. Delegates also asked about the toxicity of the iron‐oxide particles to dopaminergic neurones. Prof. Murray explained that iron‐induced toxicity had not been observed at the levels used in mouse models. She added that the primary purpose of these experiments was to optimize and validate the safety of the dopaminergic neurone biodistribution for later human use without iron‐oxide labels.

Dr. Shugo Tohyama (Department of Cardiology, Keio University School of Medicine, Japan) addressed challenges for the manufacture of ventricular cardiomyocyte‐like cells from hiPSCs for the treatment of severe cardiac failure.^14^ Dr. Tohyama focused his presentation on issues of cardiomyocyte purity and potential tumorigenicity due to residual undifferentiated iPSCs that, even at levels of 0.02% of cells, were able to form teratomas in animals and were therefore a possible risk to patients. First, he described a means of regulating the metabolism of differentiated cell preparations using a glucose and glutamine‐depleted media supplemented with lactate to eliminate undifferentiated hPSCs.^15^, ^16^ Second, he described a process to further eliminate residual undifferentiated iPSCs by treatment with an anti‐obesity drug Orlistat™ to inhibit fatty acid synthase. This inhibition induces mitochondrial apoptosis specifically in iPSCs without affecting other differentiated cells.^17^ Dr. Tohyama's work on the scale‐up aspects of manufacturing processes included the development of automated and consistent production of cardiomyocyte spheroids^18^, ^19^ which could provide sufficient cells for each patient. He also showed evidence that these spheroids showed electrical coupling with surrounding tissue, and were capable of becoming neovascularised within 12 months post‐implantation.^20^ He also revealed plans to launch a clinical trial of iPSC‐based cardiac regenerative therapy in the near future.

Dr. Shugo Tohyama was asked if he had seen any evidence of Orlistat toxicity, to which he responded that no obvious toxicity had been experienced in their observations with the levels of Orlistat used in the current manufacturing process. Furthermore, Dr. Tohyama explained that Orlistat was approved by the USFDA for use in anti‐obesity therapy and was considered safe for medical use to reduce obesity. Delegates were also interested in the mechanism of action of Orlistat and he explained that it was an inhibitor of fatty acid synthesis anti‐lipase for undifferentiated iPSCs. Delegates also asked Dr. Tohyama about the levels of residual atrial cardiomyocytes and pace‐maker cells which could impact on the final product. He reported that following differentiation, ventricular cardiomyocytes represented 95% of cells and that the subsequent metabolic selection process selected for ventricular cardiomyocytes. Further detail can be found in the tandem publication by Dr. Tohyama.^15^

Dr Jianchao Gao (Centre for Drug Evaluation, National Medical Products Administration, China) reported that the regulatory framework for development of cell therapy in China had changed significantly since 2009 with new legal governance structures (i.e., Drug Administration Law 2019), new responsible bodies and new guidance documents that would be published in English. The newly formed NMPA (a department of the Administration for Market Regulation) was responsible for commercial market authorized drugs (including cell‐based medicines), whereas the National Health Commission was responsible for regulation of hospital‐based clinical research labelled ‘medical technologies’. Finally, Dr Gao outlined the regulatory path from approval of an IND application and phase I‐III clinical followed by a Biologics Licence Application for market authorisation. He concluded by indicating that there were now many active INDs using stem cells for applications in at least seven disease areas. A full review of his presentation is included in this special edition.^21^

During the Q&A Dr Jianchao Gao was asked if the clinical studies he had described were clinician or academic researcher‐led. He responded that most studies were hospital‐based and thus fell under the National Health Commission and details of project leads did not fall under NMPAs purview. However, Dr Gao stated that all Chinese clinical studies were registered on Clinicaltrials.gov and there was no separate Chinese database of clinical studies (editorial note: Chinese hPSC‐based clinical studies are also listed in www.hescreg.eu). He also reported that he was aware that numerous companies were preparing for INDs that would fall under NMPA's jurisdiction in the future.

Dr Kapil Bharti (National Eye Institute, NIH, USA) described the high degree of complexity in the manufacture of his autologous retinal pigmented epithelial product for treatment of age‐AMD. It involved many reagents (including numerous growth factors), multiple production processes over a total production process time of 164 days. In the case of the NIH study, it had been necessary to acquire significant experience with more than 100 differentiation runs to achieve a reproducible manufacturing process where batches reliably met acceptance criteria. Crucial early in‐process controls included selection of suitable iPSC clones and maintenance of sterility of cultures. It was also vital to have highly trained staff to avoid wasted time due to contaminations or selection of poor‐quality clones. The lack of predictability for iPSC suitability using current in vitro pluripotency assays meant that for each donor 12 clones were established to provide adequate back up clones to avoid the need for repeated reprogramming. Other important in‐process quality control checks included application of HLA‐typing at various stages to exclude any cross‐contaminated cultures and therefore non‐autologous product. Dr Bharti went on to summarize the key challenges at the reprogramming (assuring robust sample traceability, need for predictive assays for pluripotency, lack of consistency in colony selection and lack of closed processes to avoid contamination) and product release stages (need to release each batch of product, critical timing between product availability and patient treatment, sterility testing results). In the light of these numerous challenges, Dr Bharti stated that it was important to establish key go/no‐go steps to avoid wasted time and resources on product that was not developing appropriately. He went on to explain the complex process of validating his autologous iPSC cell therapy IND application to progress to Phase I/IIa trial which had required demonstration of consistent production with three clones from each of three iPSC donors. The release criteria established for clinical trial included flow cytometry for cell purity, quantified cell morphology assessment, RPE‐specific gene expression, cytokine excretion (i.e., VEGF and PDGF) and phagocytosis activity. Dr Bharti also reported efforts of his group to increase the cost‐effectiveness of autologous iPSC therapy manufacture. In particular he had been pilot‐testing automated reprogramming using the Artitell (Cellino) colony picking device, which used AI‐based characterization and laser‐based elimination of poor‐quality iPSC colonies whilst holding individual batches of patient cells in a closed system until cell lines had been established. Final colony selection was also confirmed following Score‐card™ analysis of pluripotent potential. Dr Bharti saw such automated AI‐based approaches as crucial to enabling cost‐effective autologous iPSC therapies.

In Q&A, delegates expressed great interest in accessing the artificial intelligence validation tools Dr Bharti had described. He reported that there were plans to make the software available via a Cloud‐based system.

## 4 ETHICAL ISSUES IN THE DEVELOPMENT OF HPSC‐BASED MEDICINES

Prof. Rosario Isasi (University of Miami, USA) presented a view on the landscape of ethics and policy issues for the manufacture of cell‐based medicines derived from hPSCs. Prof. Isasi also emphasized the need to consider regulatory constraints, participant's interest together with IP rights through the cycle from pre‐clinical development to Phase IV post‐market surveillance. She further stated that it was important to remember that there was a range of professional networks that had already been working on these topics for hPSC‐based research and in development. In particular, she cited key documents from the International Stem Cell Initiative (ISCI) and the International Stem Cell Banking Initiative (ISCBI) documents^22^, ^23^ (see also www.iscbi.org/publications), as well as the International Stem Cell Forum ethics working party and CIRM workshops^24^ and also new ISSCR guidance on clinical translation.^25^ Isasi said it was important for product developers to be aware of any variations in international regulatory requirements and product classification. However, she also reflected on fundamental similarities in regulations, for product classification and require the manufacturer to have some understanding of the mode of action of the cell‐based therapy or product. Prof. Isasi went on to consider the principles of sharing data and bio samples and referenced the detailed protocols and code of practice developed by the EU‐funded pluripotent stem cell database called hPSCreg (www.hpscreg.eu)^23^, ^26^, ^27^ and the CIRM‐funded ‘Discuss’ project^24^ which prescribed routine review of informed consent to assure that this did not preclude derivation of cell lines and their distribution. In addition, ‘Discuss’ also outlined a framework for delivery of stem cell‐based products. Prof. Isasi went on to discuss acceptable procedures for publication of data including genotypes^28^ and the outcomes of general stem cell research.^29^, ^30^ Prof. Isasi also explained that the EU regulation and the General Data Protection Regulations impacted on hiPSC most significantly as they are directly linked to an identifiable person and thus personal data including raw genetic data, health information and other biometric data must be controlled even after leaving the EU. Thus, GDPR will impact on all non‐EU as well as EU collaborators receiving such personal data. Finally, Prof. Isasi considered the complex area of intellectual property and the areas of biomedical applications where opinions from the European Court of Justice had impacted on the patenting of hESC‐derived products.

Dr. Yaojin Peng (IOZ, CAS, China) stated that in Chinese law, the human embryo was considered to have a moral status between a human subject and an object, and is thus worthy of protection. Furthermore, hESC research was considered a worthwhile and ethically justified pursuit in the light of the potential public benefit. Dr. Peng pointed to Chinese legal precedents established in 2014 where it was concluded that in vitro fertilized human embryos had the capacity for human life and therefore such embryos could not be treated as common personal property. However, he went on to explain that Chinese patent law also changed in November 2019 to state that a patent based on the use of an in vitro embryo cultured for less than 14 days^31^ could not be refused on grounds of a violation of social morality, thus in principle permitting patentable applications of inventions regarding hESC. Dr Peng emphasized that in China, regulation of hESC research was primarily through ethical guidelines as well as general principles provided in laws.^32^ These guidelines prohibit embryo reproductive cloning and culture of human embryos beyond 14 days.

Dr. Peng went on to describe the heavy custodial sentence and fine (3 million RMB) that was applied to Dr. Juankui He who had reimplanted human embryos that were gene edited in vitro. Following this event, the Chinese regulatory authorities had established a new monitoring system for science and technology, new legislation under a Criminal Law amendment, and new Biosecurity laws. In summary, Dr. Peng reported that in China the human embryo is respected, whilst a permissive regulatory approach has been taken to facilitate stem cell research and its applications. The Chinese regulatory authorities are also continuing their efforts to address ethical issues in the field of biotechnology, in the light of new developments in science and technology. Dr. Peng has also published in tandem with this report, a more detailed consideration of the issues.^12^, ^29^, ^30^, ^33^

In open discussion with Prof. Isasi and Dr. Peng, delegates asked about the degree to which governance for individual tissues from donors fell under local or regional/national authorization. Prof. Isasi responded that this depended on whether the tissue in question was somatic or embryonic/foetal, but concluded that national law would dominate. However, she also emphasized that it was valuable to consider international regulations that may impact on stem cell products in the future. Furthermore, Prof. Isasi considered that professional best practice was especially important in areas where existing regulation was lacking. Dr. Peng added that in China two documents regulated such issues: (1) the genetic resources regulation (2019), which provides for suitable informed consent and balancing risk for donors and (2) the biomedical review of human subjects, which is under review to enhance donor protection. He emphasized that the drafting of these documents had taken into consideration the regulations in the USA and EU, including the GDPR.

Delegates were also interested as to how issues of primate and monkey embryo research and human/primate admixed embryos in particular, were managed in China. Dr. Peng said that whilst there was no specific applicable law, such research would come under the purview of the respective Institutional ethics review board before research could even begin and extended culture of admixed embryos would not be approvable.

A further concern amongst the delegates was the major challenges in assessing variation in ethical requirements in different jurisdictions. Prof. Isasi said this is fertile ground for progress especially in relation to provenance of cell lines and approval processes, where there was need for more concentrated efforts. She went on to explain that the barriers were not too significant but would require cross‐border understanding. Dr. Peng stated that adopting appropriate forms of communication is a key challenge in this area, including the need to share regulations and laws. Prof. Isasi also emphasized that good science should begin with good policy and ethics. It was generally concluded that focusing too much on interjurisdictional regulatory and ethical differences is unhelpful and it is crucial to consider levels of harmonization that already exist. Overall, the requirements for fundamental protection of donors and research integrity are similar in different regions (see Appendix 3 reference).^20^

## 5 SUMMING UP AND CONCLUSIONS

Prof. Glyn Stacey (ISCBI, UK and President's International Fellowship Initiative [PIFI] Special Expert, CAS, China) gave a summary of the key take‐home messages from the meeting and explored areas where more research and better standardization is urgently needed for hPSC‐based manufacturing. He summarized these messages under the headings of bioprocessing, product formulation/testing and standardization.

### 5.1 Bioprocessing and differentiation in manufacturing

- Understanding of cell line genetic stability and ability to differentiate into fully effective mature cell types.
- More knowledge required on cell system interactions to support identification of relevant Critical Process Parameters.
- Huge technical challenges in more complex and lengthy differentiation pathways that need measures to assure purity and reliability of product batches.
- Automation for well‐controlled reproducible manufacturing processes.
- Bioreactor scale‐up systems and cost‐effective manufacturing processes will be critical to success as many hPSC‐based products require significant expansion and product batches would need to exceed 10^9^ therapeutic cells. Effective tools and protocols for biopreservation and recovery
- Understanding of cell population interactions and impact of culture systems on cell performance.

### 5.2 Product formulation and testing

- Mode of action is often not well understood at least at the start and it will be important to have greater understanding of how cells work in the respective disease environment.
- Safety of engineered cells
- Relevant assays of tumorigenicity that are informative for patient safety
- Validation of animal models and new tools to investigate biodistribution, cell function and safety.
- New research and validated tools to enable identification of relevant and effective CQAs.
- Investigation and validation of new biomarkers and functional assays as predictive indicators of efficacy.
- Quantitative evaluation of biomarkers and functionality
- Artificial Intelligence systems will be needed to process large and complex data sets required for hPSC‐based manufacturing.
- Potency assays for different therapeutic cell types.

### 5.3 The need for standardization

- International co‐ordination is crucial and could be most effective if directed at precompetitive aspects where there could be the broadest benefit.
- International standards for development of cell lines
- Assessment of genomic stability and tumorigenicity
- Large scale culture controls
- Physical standards for cell identity*
- Consensus on optimal therapeutic cell types*
- Validated animal models for product function and patient safety
- Parallel activity to engage regulatory agencies.

Prof. Stacey went on to summarize the outcome of the Bridging session meeting (21st April, 2021) between the leadership of the Chinese Society for Stem Cell Research, the Innovation Alliance for Stem Cell Resource Centres, the human Pluripotent Stem Cell Registry (hPSCreg), the ISCBI, and the ISCI. They concurred that there were a number of aspects of common interest and thus potential opportunities exist for future collaboration.

A number of key scientific topics were identified for ongoing co‐operation between the groups and these included:

- Standards relating to the generation of high‐quality stem cells.
- Genetic stability and its relationship to the safety of cell‐based medicines.
- Safety and efficacy of cell‐based medicines derived from stem cells
- Utility and interoperability of stem cell database systems.
- International regulatory issues and best practice especially in research governance and regulatory science to advance the development of stem cell therapy.
- Training initiatives for stem cell banking, characterization and bioprocessing.
- Stem cell ethics and data sharing.

In conclusion, the Bridging meeting partners agreed to promote the international coordination and co‐operation by exchanging further information on their respective activities in the aforementioned areas and plan for follow up interactions to consider possible programmes of collaborative work.

In a personal summary, Dr. Andreas Kurtz (Fraunhofer‐IBMT, Germany and Coordinator of hPSCreg) covered a range of issues he considered to be important for future hPSC‐based therapies. In particular, he endorsed the need for international co‐ordination which had been a key element in the development of the hPSCreg project and had been mentioned by workshop panellists. In relation to research ethics, he pointed to key similarities but also differences such as the implementation of social morality in regulations of the EU and China. In terms of scientific issues for hPSC‐based medicines, Dr. Kurtz emphasized the importance of understanding the genetics of therapeutic cells. The characterization of genetic variants and target cell standards would be important, including quality control/safety testing, cell identity and genetic/epigenetic tolerance for genotypes acceptable for clinical use.

He went on to outline areas requiring active coordination and development of best practices in research ethics, including:

- Early and careful consideration of ethics issues to avoid problems in the mid‐long term
- The need to harmonize approaches to informed consent and the utility of cells and data
- The need to actively frame social morality in the light of current and potential future developments for germ cells, embryos, chimeras, bio‐data hybrids etc.

Dr. Kurtz also raised the topic of databases and the sharing of data, pointing to a number of key issues including ethical issues (e.g., anonymity, confidentiality, data misuse, inclusivity) which require the researcher to know what specific issues arise for individual cell lines. In addition, Dr. Kurtz identified that it will be important to explore what role the stem cell biobanks can play as mediators between donor and research user. Careful attention would have to be paid to regulatory issues such as data protection, data export and traceability. These issues would require practical solutions in terms of data standards for stem cells/data formats, registries of data on stem cell clinical trials with positive and negative outcomes, assays and production cell lines. Dr. Kurtz reflected on work that already started on the development of ISO standards for stem cells including stem cell data interoperability.

Prof. Martin Pera (Jackson Laboratories, USA and Coordinator of ISCI) went on to give his overall observations that great progress had been made in stem cell research in recent years and in particular recognized the leadership shown by Chinese scientists in the 2000s to advance international coordination in the development of pluripotent stem cell therapies. He saw great opportunity and will for there to be constructive collaboration with groups such as ISCI, ISCBI and the new ISSCR initiatives in the area of stem cell manufacturing. He maintained that such interactions will be crucial to provide authoritative debate to back the development of standards in this area. Prof. Pera also commented on the consistent messages throughout the workshop sessions that had emphasized the importance of science to underpin the area of stem cell‐based manufacturing. In conclusion, Prof. Pera identified the crucial importance of support for the area between basic research and clinical practice which would significantly benefit from new wet laboratory collaboration on an international basis.

Finally Prof. Qi Zhou (CAS, China), the initiator of the PSConf 2021 meeting, concluded that the levels of scientific achievement presented at the meeting were very exciting and the discussions were of a very high quality. He was glad to see the collaborative spirit that delegates and speakers had brought to the meeting. Prof. Zhou also noted that the PSConf meeting series aims to bring global scientists, manufacturers, product developers and regulators together to brave the new challenges of hPSC translation in unison, and envision the future of hPSC translation together. Therefore, he hoped the meeting series will help to forge broader collaborations to advance the frontiers in this exciting field of stem cell and regenerative medicine.

He encouraged all delegates and speakers to further build upon the interactions which the meeting had initiated, and he looked forward to welcoming delegates from all countries to future meetings.

## FUNDING INFORMATION

Glyn N. Stacey was supported by the Chinese Academy of Sciences President's International Fellowship for Special Experts (2018FSB0009). Baoyang Hu was supported by the National Key Research and Development Program of China (2017YFE0122900). Qi Zhou was supported by the National Natural Science Foundation of China (31621004) and the Strategic Priority Research Program of the Chinese Academy of Sciences (XDA16030000).

6

## Data Availability

There is no data applicable to this manuscript.

## APPENDIX 1 INFORMATION ON THE ORGANIZATIONS INVOLVED IN PLANNING PSConf 2021

### A.1 Institute of Zoology, Chinese Academy of Sciences

The Institute of Zoology (IOZ) of Chinese Academy of Sciences (CAS, http://english.ioz.cas.cn/) is a Beijing based institute that is dedicated to improve the quality of human life by pursuing research excellence in stem cell and reproduction, development and ageing, and evolution and ecology. There are currently 420 staff, including 75 principal investigators. One of the most ground‐breaking achievements include generation of the first iPSC‐derived mouse. Both the National Stem Cell Resource Centre and the Institute for Stem Cells and Regeneration (see below) originated from this institute.

### A.2 Institute for Stem Cell and Regeneration, Chinese Academy of Sciences

Institute for Stem Cell and Regeneration (ISCR), Chinese Academy of Sciences (CAS; http://iscr.ac.cn/en/) integrates scientific teams and high‐level research platforms under CAS and actively explores new models of intra‐ and international collaboration, to promote stem cell R&D and industrialization. Aiming to accelerate the clinical translation, the ISCR‐CAS focuses on the frontiers of the following areas: (1) ageing and the relevant disorders; (2) innovative biotechnologies; (3) reproductive biology; (4) cell drugs and biotherapy; (5) stem cell and regenerative medicines; and (6) organ engineering (http://iscr.ac.cn/en/).

### A.3 Beijing Institute for Stem Cell and Regenerative Medicine

Building on the success of ISCR CAS, the Beijing Institute for Stem Cell and Regenerative Medicine (BISCRM, http://iscr.ac.cn/en/about/biscrm) was founded in 2020 as a joint strategic innovation initiative of CAS and the Beijing Municipal Government. The BISCRM conducts original research, targeting unmet national needs and global scientific frontiers in stem cell and regenerative medicine.

### A.4 International Stem Cell Forum

The International Stem Cell Forum (ISCF) was originally formed in 2002 by Professor George Radda of the UK Medical Research Council, to promote international collaboration towards the development of stem cell‐based therapies. It has been chaired and revitalized by Professor Qi Zhou, Director of the ISCR CAS, since 2014, with earmarked resource from key funders including CAS and Chinese Society for Stem Cell Research (CSSCR). The ISCF's mandate is to promote the development of international scientific coordination on issues crucial to our understanding of pluripotent stem cells and their therapeutic applications. ISCF will continue to be provide multidisciplinary support for translational research on pluripotent stem cells.

### A.5 Chinese Society for Stem Cell Research

Founded in 2007, the CSSCR is now the largest society for stem cell and regenerative medicine in China, with 52 directors and over 2000 members from all over China. The participants include members from academia, hospitals, industry, and government. The responsibilities of CSSCR are to promote academic exchanges, popularize science education, develop international cooperation, recommend outstanding talents, and provide advice on policy making in the field of stem cell research.

### A.6 National Stem Cell Resource Centre Innovation Alliance

Formed in 2019 under the leadership of Prof Qi Zhou, this National Alliance is formed in partnership with nine biobanking institutions across China. The Alliance aims to carry out collaborative research on biobanking and to create a biobank resource network to deliver tools and services for all types of researchers and biobankers, for example, certification and accreditation, training and education, and adoption of best practice standards.

### A.7 National Stem Cell Resource Centre

The National Stem Cell Resource Centre of China originated from the Beijing Stem Cell Bank (IOZ CAS since 2007) and was officially awarded its new name in 2019. It is a national resource centre that adheres closely to international standards, and provides resources and supports for basic research and future clinical applications of human stem cells. A major objective will also be the establishment of an International Stem Cell Bank hub and training centre which can support the accreditation of stem cell banks and their staff and qualification of pluripotent stem cells for clinical use.
